# Johnson County Medical Society

**Published:** 1893-07

**Authors:** 


**A Meeting** of the Johnson County Medical Society was held on the 13th day of June, at Cleburne, in the office of Dr. T. C. Osborn, according to the call of the President.

The Society, dormant for some time, was re-vivified and the following officers were elected: President—Dr. J. L. Wagley; First Vice-President—Dr. J. M. Towns; Second Vice-President —Dr. W. M. Yater; Secretary and Treasurer—Dr. T. C. Osborn, for one year.

Drs. J. L. Wagley, T. C. Osborn and G. B. Colby were selected as the Board of Censors, for three years.

